# One-Pot Click Access to a Cyclodextrin Dimer-Based Novel Aggregation Induced Emission Sensor and Monomer-Based Chiral Stationary Phase

**DOI:** 10.3390/s16121985

**Published:** 2016-11-24

**Authors:** Xiaoli Li, Rui Zhao, Xiaoying Tang, Yanyan Shi, Chunyi Li, Yong Wang

**Affiliations:** 1 Tianjin Key Laboratory of Molecular Optoelectronic Science, Department of Chemistry, School of Science, Tianjin University, Tianjin 300072, China; lixiaolitju@163.com (X.L.); zhaoruisummer@163.com (R.Z.); tangxiaoying@tju.edu.cn (X.T.); yyshi@tju.edu.cn (Y.S.); 2 Collaborative Innovation Center of Chemical Science and Engineering, Tianjin 300072, China; tianqing@tju.edu.cn

**Keywords:** click, cyclodextrin, aggregation induced emission, chiral stationary phase

## Abstract

A ‘two birds, one stone’ strategy was developed via a one-pot click reaction to simultaneously prepare a novel cyclodextrin (CD) dimer based aggregation induced emission (AIE) sensor (AIE-DCD) and a monomer based chiral stationary phase (CSP-MCD) for chiral high performance liquid chromatography (CHPLC). AIE-DCD was found to afford satisfactory AIE response for specific detection of Zn^2+^ with a detection limit of 50 nM. CSP-MCD exhibits excellent enantioseparation ability toward dansyl amino acids, where the resolution of dansyl amino leucine reaches 5.43.

## 1. Introduction

Cyclodextrins (CDs) are known to embody a unique hydrophobic cavity which is able to encapsulate different kinds of guest molecules and a hydrophilic surface originating from the hydroxyl moieties on the rims [1,2,3]. Benefitting from its special conformation, CDs have been widely used in many research areas such as chromatography, catalysis, drug delivery, water purification, and sensing [4,5,6,7,8]. By far, although there a large amount of CD functional materials have appeared, most of them are based on random substitution reactions on CD rims. Owing to the close reaction activity of CD hydroxyls and the modest solubility of CDs in organic solvents, it is difficult to obtain CD derivatives with high purity and well-defined structures only by chromatography purification. Hence, development of facile approaches to prepare structurally well-defined CD functional materials still remains a challenging art.

Click chemistry, a concept established by Sharpless et al., offers benign reaction conditions, simple purification methods, high efficiency, and good selectivity [9]. The Cu(I) catalytic cycloaddition between terminal alkyne and azide (CuAAC) has become the prime click reaction type for not only modularly linking functional units but also synthesizing the triazole moiety which is an important coordinating precursor [10,11,12,13]. Our previous efforts have been dedicated to the use of CuAAC to fabricate structurally well-defined CD-based chiral stationary phases (CD-CSPs). Different mono-azido-CD derivatives have been synthesized and anchored onto alkyne functionalized silica surfaces to afford a series of functional CD-CSPs which can be applied in chiral liquid chromatography (LC) and capillary electrochromatography (CEC) for enantioseparation of a large variety of chiral compounds [14,15,16,17,18,19].

On a separate note, in recent years, the phenomenon of aggregation-induced emission (AIE) is attracting increasing attention in many areas, especially sensing and bio-imaging [20,21,22]. Molecules with AIE activity usually have no fluorescent property in solution due to the non-radiative decay caused by intramolecular motions, whereas they become highly emissive upon aggregation since the intramolecular motions are restricted [23,24,25]. Drawing support from the versatile CuAAC, very recently, our group first reported the synthesis and application of a novel triazole-bridged AIE-CD monomer sensor which affords excellent selective turn-on fluorescence response to Cd^2+^ with a low detection limit of 0.01 μM [26]. This finding reveals the great potential of triazole-bridged AIE-CD in chemical and biological sensing. Therefore, it is very necessary to extend our former study to synthesize triazole-bridged AIE-CDs like dimers, trimers, even tetramers to conduct further study in this new research direction. However, due to the similar properties of the reactant N_3_-CD and target product (Figure S1) and their modest solubility in organic solvents, it is very hard to obtain pure target product by simple chromatography purification.

Considering our previous efforts in the preparation of CD-CSPs, it is possible that the N_3_-CD residue in the crude product of AIE-CDs can be removed by subsequent click reaction with alkyne functionalized silica to afford triazole-bridged CD-CSP as a by-product. With this idea, in this work, we would like to develop a ‘one-pot’ click procedure to obtain two structurally well-defined functional CD materials simultaneously: an AIE-CD dimer sensor and a CD monomer-CSP. As far as we know, there are very few reports describing the ‘two birds, one stone’ strategy for the preparation of structurally well-defined CD materials. This approach is believed to provide a facile way to obtain novel CD derivatives and solid-based CD materials with a well-defined structure [27,28].

## 2. Materials and Methods

### 2.1. Materials

4-Hydroxy diphenyl ketone, propargyl bromide and tetrabutyl ammonium bromide were purchased from HEOWNS (Tianjin, China). Sodium azide, CuSO_4_·5H_2_O, and pyridine were purchased from Tianjin Chemical Regents (Tianjin, China). Tetrahydrofuran (THF), dimethylsulfoxide (DMSO), toluene, and dichloromethane were purchased from JiangTian chemical reagents (Tianjin, China). Ascorbic acid sodium salt, *N*,*N*-Dicyclohexylcarbodiimide, propiolic acid, and 3-aminopropyltriethoxysilane were provided by Energy-Chemical (Shanghai, China). Kromasil spherical silica gel (5 μm, 100 Å) was purchased from Eka Chemicals (Bohus, Sweden). Mono-6A-deoxy-(p-tosylsufonyl)-β-cyclodextrin (TsO-CD) and alkynl modified silica was synthesized according to the reported procedure. THF and toluene were distilled over Na/benzophenone prior to use. Other chemicals were of analytical grade and used without further purification. The metal salts used for study were PbCl_2_, ZnCl_2_, CoCl_2_, FeCl_3_, MgCl_2_, CaCl_2_, KCl, CuCl_2_, CdCl_2_, NaCl, NH_4_Cl, Al(NO_3_)_3_, and AgNO_3_. Deionized (DI) water was used in the experiments.

### 2.2. Synthesis of TPE-Triazole-CD and CSP via ‘One-Pot’ Click Chemistry

Mono-(6-azido-6-deoxy)-β-CD (4.3 g, 3.69 mmol) was added to a solution of TPE-alkynl (0.54 g, 1.23 mmol) in DMF (55 mL) followed by addition of CuI(PPh_3_) (60.2 mg, 0.13 mmol) in one portion. The reaction mixture was stirred at 90 °C for 24 h under nitrogen atmosphere followed by addition of alkynl modified silica (4.35 g) and kept for stirring for another 24 h. The reaction mixture was filtered, the obtained solid was washed with DMF, ethanol, and acetone for 8 h before vacuum drying to afford the triazole-CD-CSP. The combined organic fractions were dried over MgSO_4_. The solvents were removed and the crude product was purified by flash chromatography using silica gel with water and acetonitrile (1:2, *v*/*v*) as the eluent. Yellow solid was obtained to afford the TPE-2triazole-2CD.

### 2.3. Instrumentation

^1^H and ^13^C-NMR were recorded on a Bruker ACF400 (400 MHz) supplied by Bruker Biospin (Fällanden, Switzerland) in deuterated chloroform (CDCl_3_), dimethylsulfoxide (DMSO-d_6_), or D_2_O using tetramethylsilane (TMS) (δ = 0) as internal reference. Fourier-transform infrared (FTIR) spectra were collected on an AVATR360 supplied by Thermo Nicolet (Madison, WI, USA). Mass spectra were recorded on LCQ Deca XP MAX system (Thermo Fisher, Waltham, MA, USA). High resolution mass spectra (HR-MS) were measured on a miorOTOF-QII supplied by Bruker Daltonics (Billerica, MA, USA). The fluorescence spectra were taken on a Cary Eclipse fluorescence spectrophotometer supplied by Varian (Palo Alto, CA, USA) at room temperature. Particle size distribution analysis was carried out on a Delsa NaNo C provided by Beckman Coulter (Indianapolis, IN, USA). TEM-EDX was taken on a FEI Tecnai G2 F20 TEM (Eindhoven, The Netherlands) with EDX equipment at an accelerating voltage of 200 kV. The morphologies of aggregates of compounds at the nano-scale were determined on a JEM-2100F supplied by Japan at an accelerating voltage of 200 kV. TEM images were collected on copper mesh.

## 3. Results and Discussion

**Scheme 1:**
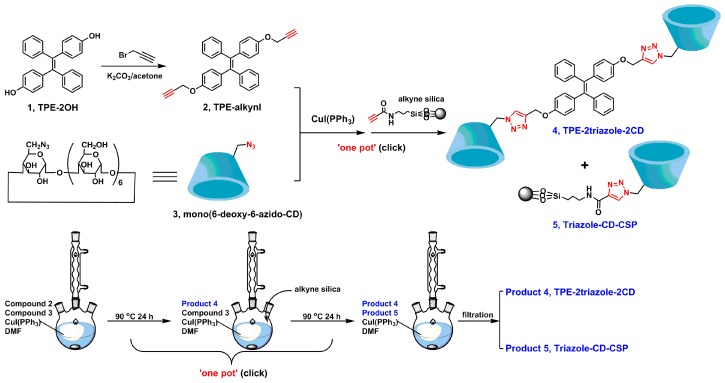
‘One-pot’ click synthetic pathway of the AIE-CD dimer sensor (TPE-2triazole-2CD) and monomer CD-CSP (Triazole-CD-CSP).

The designed synthetic pathway is outlined in Scheme 1. In contrast to aggregation-caused quenching (ACQ) of traditional fluorophore, TPE derivatives can exhibit strong emission when they are aggregated or in the solid state [29,30,31]. At the same time, tetra-phenyl ethylene (TPE) is a typical fluorophore which processes the advantages of facile synthesis and easy structural modification; numerous functional groups can be attached to it through simple reactions, so it was chosen as the AIE fluorescence core in this study. To implement the one-pot click process for the preparation of two materials, three precursors (TPE-2alkyne, mono-6-azido-CD, and alkyne silica) should be prepared initially. The click precursor TPE-2alkynl (compound **2**) was prepared by reacting TPE-2OH (synthesized via McMurry coupling reaction according to the reported methods with a yield of 76% [32]) with propargyl bromide in a basic environment and characterized by ^1^H-NMR (Figure S2 ESI^†^). The precursors mono-6-azido-CD (compound **3**) and alknyl silica were synthesized following our previous methods [33,34].

The ‘one-pot’ click procedure was thereafter carried out by initially reacting 2 with 3 in excess amount in N,N-Dimethylformamide (DMF) catalyzed by CuI(PPh_3_) [35,36,37,38] followed by addition of alkyne functionalized silica to further continue the click reaction. After completion of the reaction, the two products can be separated by simple filtration. The pure sensor TPE-2triazole-2CD (compound **4**) can be obtained by concentrating the filtrate and purified by flash chromatography and the Triazole-CD-CSP can be afforded by washing the filter-cake with DMF, ethanol, and acetone successively followed by vacuum drying. The correct structure and the satisfied purity of the TPE-2triazole-2CD can be evidenced by ^1^H and ^13^C-NMR spectra of compound **5** (Figures S3 and S4 ESI^†^) and the sole peak (*m*/2*z* = 1380.9721 measured. 1379.965 calculated) in its HR-MS spectrum (Figure S5 ESI^†^). The increase of carbon content from alkynl silica to CD-CSP (Table S1 ESI^†^) suggests the surface monomer CD concentration on silica is around 0.58 μm/m^2^ (a very high surface loading for CD-CSPs) calculated according to the commonly used equation (Table S1 ESI^†^). The above results indicate that the designed ‘one-pot’ click approach has successfully utilized to prepare two structurally well-defined CD materials.

**Figure 1:**
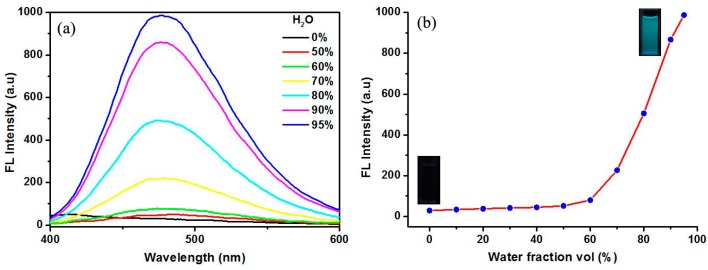
(**a**) Fluorescence emission spectra for solution of TPE-2triazole-2CD (50 μM) in different ratios of DMSO/H_2_O (λ_ex_ = 330 nm, ex/em slits = 5/5); (**b**) Fluorescence response of the sensor (50 mM) in different solvent compositions of H_2_O/DMSO at 475 nm (λ_ex_ = 370 nm).

To further approve the effectiveness of the one-pot click approach, we firstly evaluated the AIE properties of the novel sensor. As TPE-2triazole-2CD has low solubility in water, we chose H_2_O and DMSO mixtures to investigate its AIE feature initially. Figure 1 depicts the AIE characteristic of the AIE-CD dimer. As shown, the novel AIE-CD sensor does not show fluorescent character in its DMSO solution excited at 330 nm. With the increase of water fraction from 0% to 60%, the FL intensity of TPE-2triazole-2CD is only slightly enhanced, however, a critical point appears at 60% water fraction where a sharp FL intensity increase takes place (emission at 475 nm) and the FL intensity reaches 1000 a.u. when water fraction is raised to 95%. As it was proved by 2D-NMR in our previous study, this typical AIE feature should be attributed to the formed rigid structure caused by the aggregation. The decreased solubility of the AIE-CD in the aqueous mixture with high water content induces aggregate formation. In the aggregation state, the intramolecular rotation of the benzyl ring of the AIE-CD was restricted, may prevent the nonradiative pathway, resulting in the enhancement of emission.

**Figure 2:**
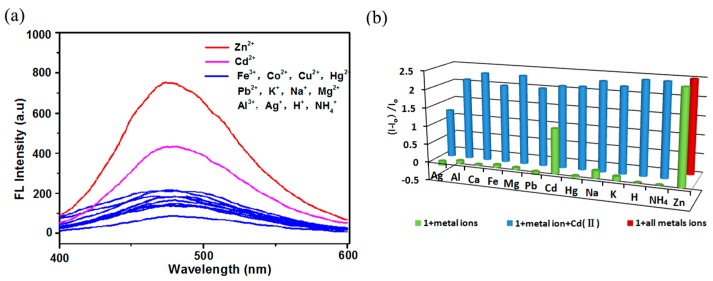
(**a**) The FL intensity of TPE-2triazole-2CD (50 μM) in DMSO/H_2_O (2/3, *v*/*v*) in the presence of 5 equivalent of different metal ions; (**b**) Fluorescence response of the sensor (50 μM) to 0.5 equivalent of metal ions (the green bar) and to the mixture of 0.5 equivalent of other metal ions with 0.5 equivalent of Zn^2+^ (the blue bar) and the fluorescence response of TPE-2triazole-2CD toward Zn^2+^ + all metal ions (the red bar) (λ_ex_ = 330 nm, λ_em_ = 478 nm).

The existence of abundant H-bonding and coordinating sites on TPE-2triazole-2CD allows for the usage of the novel AIE-CD dimer as turn-on florescence chemosensors. To investigate further, the following metal ions assay was performed under the critical turn-on florescence conditions (50 mM, 60% water fraction). Figure 2a depicts the fluorescence response of AIE-CD dimer towards different cations such as H^+^, NH_4_^+^, Na^+^, K^+^, Ag^+^, Al^3+^, Ca^2+^, Fe^3+^, Mg^2+^, Pb^2+^, Zn^2+^, Cd^2+^, and Hg^2+^. It is interesting to find that the turn-on fluorescence of TPE-2triazole-2CD can be selectively triggered by both Zn^2+^ and Cd^2+^. Benefitting from the well-designed structure, the CD hydroxyl moieties and triazole nitrogen atoms can work synergistically in coordinating Zn^2+^ and Cd^2+^ inducing the aggregation of TPE-2triazole-2CD to form a rigid structure hence inducing the fluorescence. Since the FL intensity of Zn^2+^ is almost two times higher than Cd^2+^, the TPE-2triazole-2CD is more suitable for the usage as a Zn^2+^ chemosensor. The specific fluorescence response towards Zn^2+^ over a series of interfering metal ions such as Ag^+^, Al^3+^, Ca^2+^, Zn^2+^, Fe^3+^, Mg^2+^, Pb^2+^, Hg^2+^, Cd^2+^ and an ion mixture indicates that the novel TPE-2triazole-2CD can serve as an excellent Zn^2+^ turn-on fluorescence chemosensor (Figure 2b).

**Figure 3:**
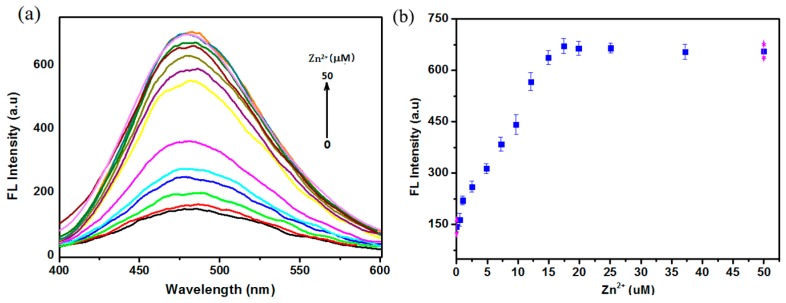
(**a**) Fluorescence emission spectra change of TPE-2triazole-2CD exposed to Zn^2+^ of various concentrations: 0, 0.5, 1, 2.5, 5, 7.5, 10, 12.5, 15, 17.5, 20.0, 25, 37.5, 50 μM from bottom to top (DMSO/H_2_O = 2/3, *v*/*v*); (**b**) Fluorescence titration curve of TPE-2triazole-2CD with Zn^2+^ in DMSO-H_2_O (2/3, *v*/*v*) solution.

**Figure 4:**
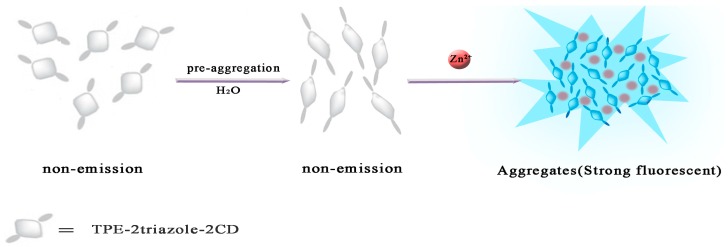
Possible proposed mechanistic pathway for sensing Zn^2+^ ion-based on Aggregation Induced Emission.

In order to figure out the binding stoichiometry between Zn^2+^ and the sensor, fluorescence titration was conducted in this study (Figure 3). It was discovered that the FL intensity of TPE-2triazole-2CD is almost linearly accentuated by increasing the Zn^2+^ concentration from 0 to 17.5 μM and levels off after Zn^2+^ concentration reaches 17.5 μM. As the TPE-2triazole-2CD concentration was 50 μM, the fluorescence titration results and the Job’s plot (Figure S6 ESI^†^) indicate the binding stoichiometry between TPE-2triazole-2CD and Zn^2+^ should be 3:1. In order to conduct further investigation on the aggregation of TPE-2triazole-2CD upon the addition of Zn^2+^, particle size distribution analysis (Figure S7 ESI^†^) and transmission electron microscopy (TEM) (Figure S8 ESI^†^) measurements were also performed. It was found that the particle size of TPE-2triazole-2CD in DMSO/H_2_O was significantly increased by addition of Zn^2+^ and the aggregation can participate from the solution at higher sensor concentration which is clearly visible without excitation by UV (Figure S9 ESI^†^). The remarkably changed morphology of TPE-2triazole-2CD before and after addition of Zn^2+^ reveals the diversification of the micro-environment on the addition of Zn^2+^. The above results indicate that the sensing process of TPE-2triazole-2CD towards metal ions should be realized by the binding caused aggregation to turn on its fluorescence based on the restricted intramolecular rotation (RIR) mechanism. We have already known that water can form a bridge between the hydroxyl groups of adjacent molecules of CD to induce aggregation [39]. By controlling the water fraction and sensor concentration, the pre-aggregation effects and the rigid structure can provide appropriate conditions for the formation of complex between TPE-triazole-CD and Zn^2+^ (Figure 4). Since the hydrophobic cavity of CD can accommodate various guest molecules forming stable complex, the novel TPE-2triazole-2CD has great potential to act as a biosensor, which is still under investigation.

**Figure 5:**
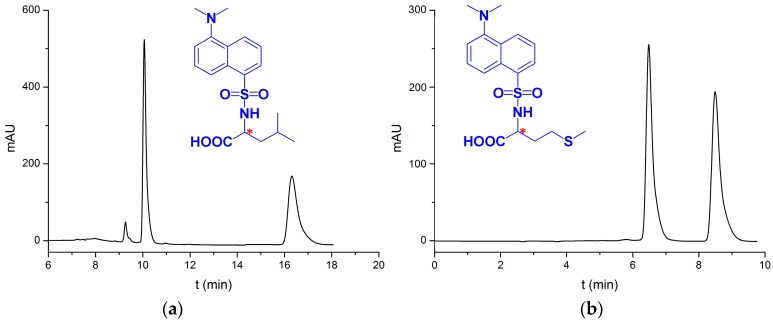
Representative chromatograms on Triazole-CD-CSP. Conditions: 1% TEAA buffer (pH 4.11)/MeOH (50/50 *v*/*v*); flow rate = 0.8 mL/min. (**a**) Dansyl amino leucine; (**b**) Dansyl amino methionine.

To adequately appraise the ‘one-pot’ click strategy, the effectiveness the other important product Triazole-CD-CSP should also be evaluated separately from TPE-2triazole-2CD. As shown in Figure S10 ESI^†^, after the ‘one-pot’ click reaction, the silica particles still keep good morphology, indicating that this method affords a mild reaction environment for the preparation of CD-CSPs. To evaluate the CSP’s separation ability, the CSP was packed into a stainless-steel column (150 mm × 4.6 mm I.D.) and subjected to high performance liquid chromatography (HPLC) for the enantioseparation of various enantiomer pairs such as dansyl amino acids and flavonoids in reversed-phase mode. The separation results are listed in Table S2 ESI^†^. Some representative chromatograms obtained by optimizing the separation conditions are illustrated in Figure 5. As it is shown, the resolution of dansyl amino leucine and dansyl amino methionine can be well resolved (Resolution = 5.43 and 2.62 respectively) using triethyl ammonium acetate (TEAA) buffered at pH 4.1 and methanol as the mobile phase (MP). For the neutral analytes flavanone and 4’-hydroxyflavanone, Triazole-CD-CSP also affords satisfactory separation performance. CDs and their derivatives have been widely used as chiral selectors in enantioselective chromatography due to their natural chirality and the ability to form inclusion complex with molecules via the unique hydrophobic cavity. The better resolution of 4’-hydroxyflavanone may be ascribed to the H-bonding formed between flavanone –OH and CD rims. The above results indicate that the resolving ability of the current Triazole-CD-CSP is favorably comparable to our previously reported CCN-CSP with similar structure, which affirms the effectiveness of the ‘one-pot’ click reaction in the preparation of the two CD functional materials.

## 4. Conclusions

In conclusion, ‘one-pot’ click strategy provides an effective and versatile approach to the preparation of cyclodextrin dimer-based novel aggregation induced emission sensor (TPE-2triazole-2CD) and monomer-based chiral stationary phase (Triazole-CD-CSP) simultaneously. TPE-2triazole-2CD shows a highly selective turn-on florescence response towards Zn^2+^ in neutral environments and is expected to act as a good biosensor owing to its complexation ability and potential biocompatibility. Triazole-CD-CSP affords comparable enantioselectivity compared to the CCN-CSP previously prepared solely via Cu(I) catalyzed 1,3-dipolar cycloaddition reaction (click chemistry). This work presents a good strategy to design and construct novel derived CD molecules and solid support CD functional materials with well-defined structures.

## Supplementary Materials

The supplementary materials are available online at http://www.mdpi.com/1424-8220/16/12/1985/s1.
