# Supplementary material for: One-Pot Click Access to a Cyclodextrin Dimer-Based Novel Aggregation Induced Emission Sensor and Monomer-Based Chiral Stationary Phase
Source: Sensors (Basel). 2016 Nov 24;16(12):1985. doi: 10.3390/s16121985 (PMC5190966; doi:10.3390/s16121985)
Supplement: Supplementary file 1 [file sensors-16-01985-s001.pdf]

Supplementary Materials: One-Pot Click Access to Cyclodextrin Dimer Based Novel Aggregation Induced Emission Sensor and Monomer Based Chiral Stationary Phase

Xiaoli Li, Rui Zhao, Xiaoying Tang, Yanyan Shi, Chunyi Li and Yong Wang

1. Synthesis and Characterization

1.1. Synthesis of 1,2-Bis(4-hydroxyphenyl)-1,2-diphenylethene

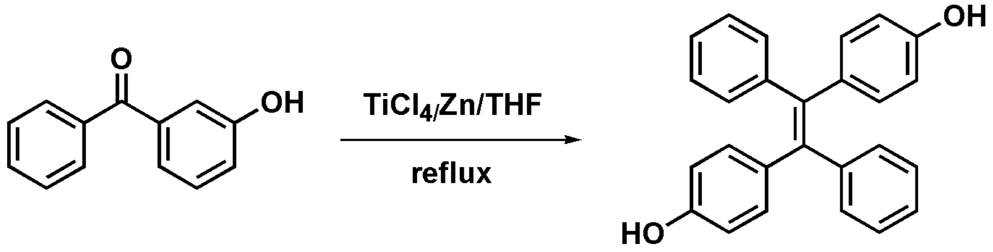

Under nitrogen atmosphere, a three-necked flask equipped with a magnetic stirrer was charged with zinc powder (1.084 g, 7.8 mmol), 4-hydroxydiphenyl ketone (0.991 g, 5 mmol) and 40 mL dry THF. The mixture was cooled to between −5 and 0 °C, and $TiCl_4$ (1.68 mL, 15 mmol) was slowly added by a syringe with the temperature kept below 0 °C. The suspending mixture was warmed to room temperature and stirred for 0.5 h, then heated to reflux until the carbonyl compounds were consumed completely (monitored by TLC). The reaction was quenched with 10% K_2CO_3 aqueous solution and extracted with CH_2Cl_2. The organic layer was collected. After solvent evaporation, the crude product was purified on a silica gel column using ethyl acetate/petroleum ether (1:5, *v*/*v*) as eluent to afford the desired product as a white powder in a yield of 76%. ^{1}H-NMR (400 MHz, $CDCl_3$): δ (ppm) 7.11–7.02 (m, 10H), 6.88 (t, 4H, *J* = 9.2, 13.6 Hz), 6.56 (d, 4H, *J* = 8.4, 12.0 Hz). ^{13}CNMR (400 MHz, $CDCl_3$): δ (ppm) 154.1, 144.2, 139.7, 135.5, 132.8, 131.5, 127.8, 126.3, 114.7. ESI (+)-MS: calcd. for $C_{26}H_{20}O$: 364 [M]; found 365.9 $[M + H]^+$; IR (ν, KBr): 1246, 1441, 1516 and 3345 cm^{-1}.

1.2. Synthesis of TPE-Alkynl

The mixture of compound **1** (1.53 g, 4.2 mmol), propargyl bromide (1.5 mL, 12.3 mmol), K_2CO_3 (4.53 g, 32.7 mmol) and NBu_4Br (21 mg, 0.059 mmol) in acetone (25 mL) was refluxed overnight under nitrogen. The mixture was then filtered and dried over anhydrous $MgSO_4$. After the solvent evaporation, the crude product was purified by a silica gel column using ethyl acetate/petroleum ether (1:5, *v*/*v*) as eluent affording a light yellow syrup in a yield of 95%. ^{1}H-NMR (400 MHz, $CDCl_3$): δ (ppm) 7.02–6.94 (m, 10H), 6.84 (m, 4H), 6.62 (m, 4H), 4.50 (m, 4H), 2.43 (m, 2H). ^{13}CNMR (100 MHz, $CDCl_3$): δ (ppm) 156.3, 144.2, 140.0, 137.4, 132.7, 131.6, 127.9, 126.5, 114.3, 78.8, 75.8, 55.9. ESI (+)-MS: calcd. for $C_{32}H_{24}O_2$: 440.5 [M]; found 441.3 [M + H]+.

1.3. Synthesis of Mono-(6-azido-6-deoxy)-β-CD

NaN_3 (1.26 g, 19.4 mmol) was added to a solution of TsO-CD (5 g, 3.88 mmol) in water (50 mL). The reaction mixture was stirred at 80 °C for 12 h, then the clear solution was poured in acetone (300 mL). The white precipitate was isolated by filtration and washed with acetone to afford mono-(6-azido-6-deoxy)-β-CD with a yield of 97%. ^{1}H-NMR (DMSO-d6): δ (ppm): 5.9–5.6 (14H), 5.0–4.8 (7H), 4.6–4.4 (6H), 3.8–3.2 (42H). IR (ν, KBr): 3357, 2928, 2107, 1032 cm^{-1}; ESI (+)-MS: calcd. for $C_{42}H_{70}O_{34}N_3$: 1160 [M]; found 1161.1 [M + H]+.

1.4. Synthesis of N-3(triethoxysilyl)propyl-2-propynamide

The mixture of 3-aminopropyltriethoxysilane (2.4 g, 10.85 mmol) and propiolic acid (0.87 g, 11.93 mmol) in anhydrous CH_2Cl_2. Then *N*,*N*-Dicyclohexylcarbodiimide (2.46 g, 12.0 mmol) was added. The mixture was stirred at room temperature for 1 h. Upon the completion of reaction, the dicylohexylurea formed was filtrated off and the excess of propiolic acid was removed by co-evaporation with dry toluene to afford light yellow oil. IR (ν, KBr): 3248, 2934, 2857, 2104, 1653, 1531, 1451, 1208, 737 cm^{-1}.

1.5. Synthesis of Alkynl Modified Silica

Silica (4 g, 5 µm) was added to a solution of alkynyl coupling agent (1 mL) in anhydrous toluene (15 mL). The suspending mixture was heated at reflux for 2 h. The solvent was evaporated to half of its volume and another portion of anhydrous toluene (15 mL) was added again and the reaction system was refluxed for another 2 h. The reaction mixture was then filtered and unreacted impurities in the crude product were removed by rinsing with acetone in Soxhlet extractor to afford light yellow powder. IR (ν, KBr): 3442, 2121 (alknyl), 1646, 1100, 471 cm^{-1}.

2. Figures and Tables

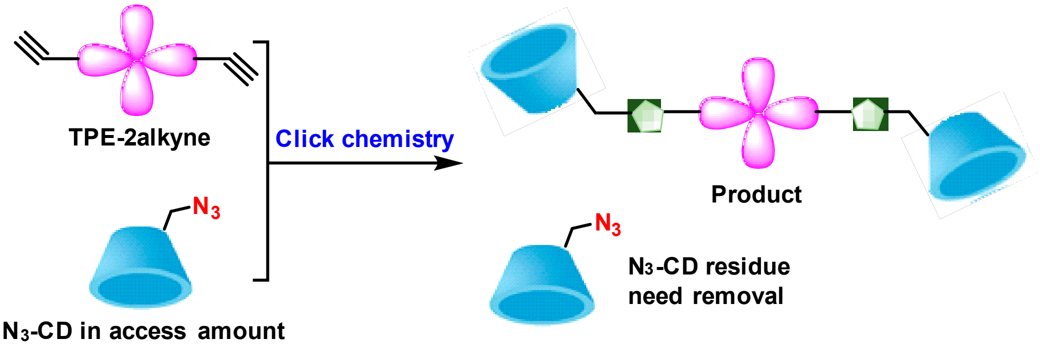

Figure S1. Sketch click reaction between TPE-2alkyne and N_3-CD.

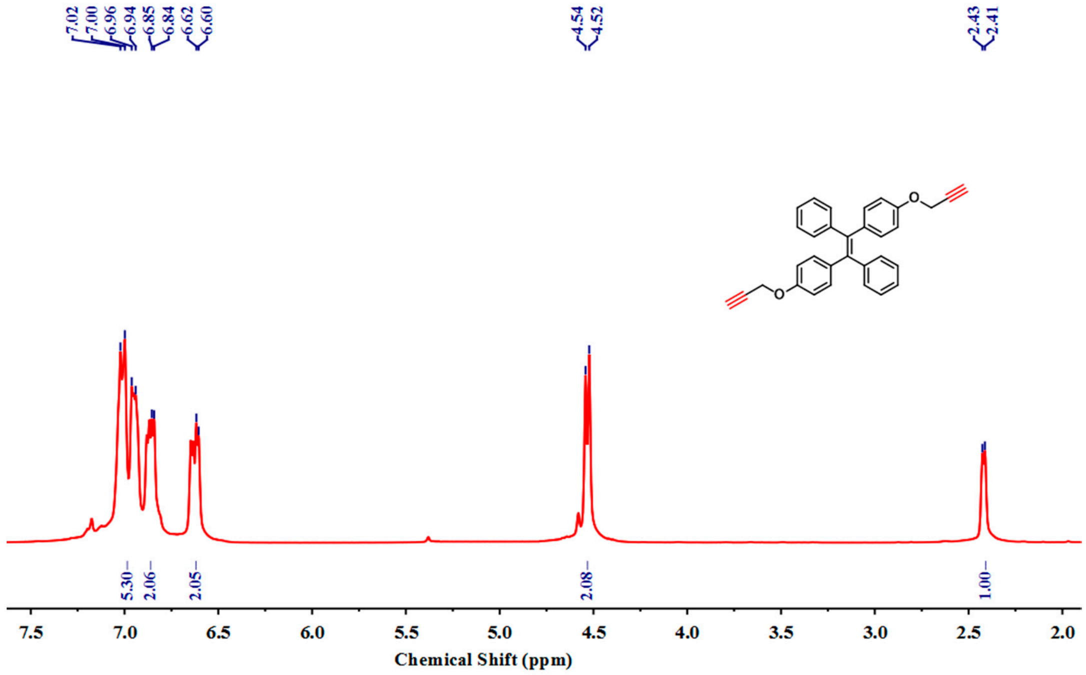

Figure S2. ^{1}H-NMR of TPE-alkyne in $CDCl_3$.

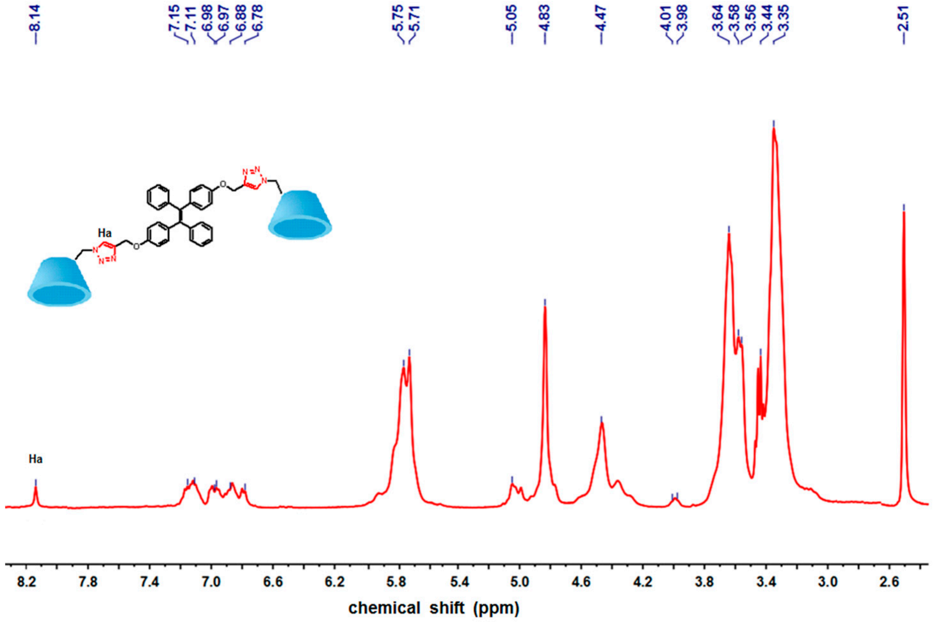

Figure S3. ^{1}H-NMR of TPE-triazole-CD in DMSO-d_6.

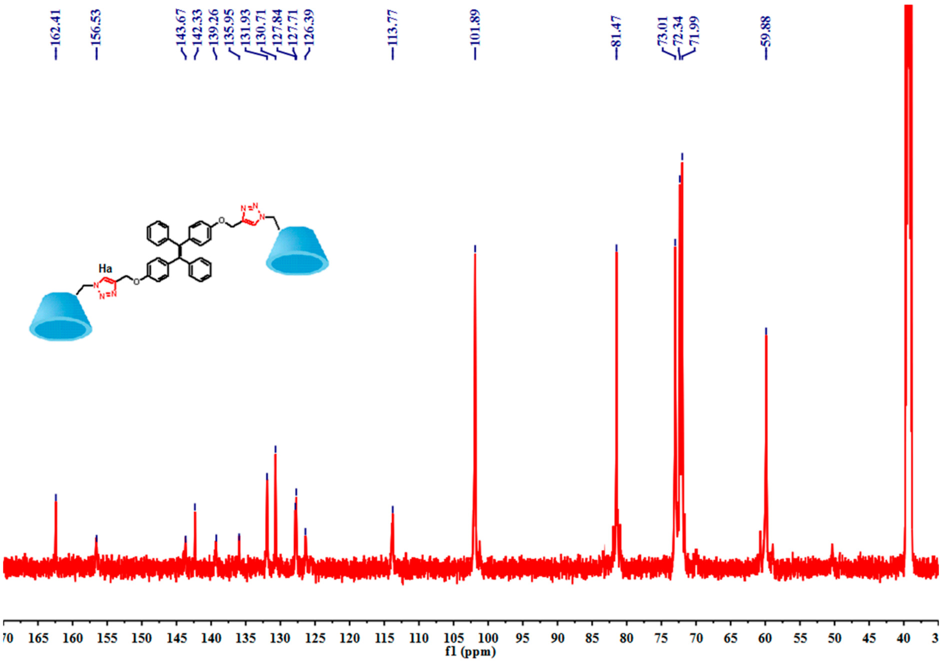

Figure S4. ^{13}C-NMR of TPE-triazole-CD in DMSO-d_6.

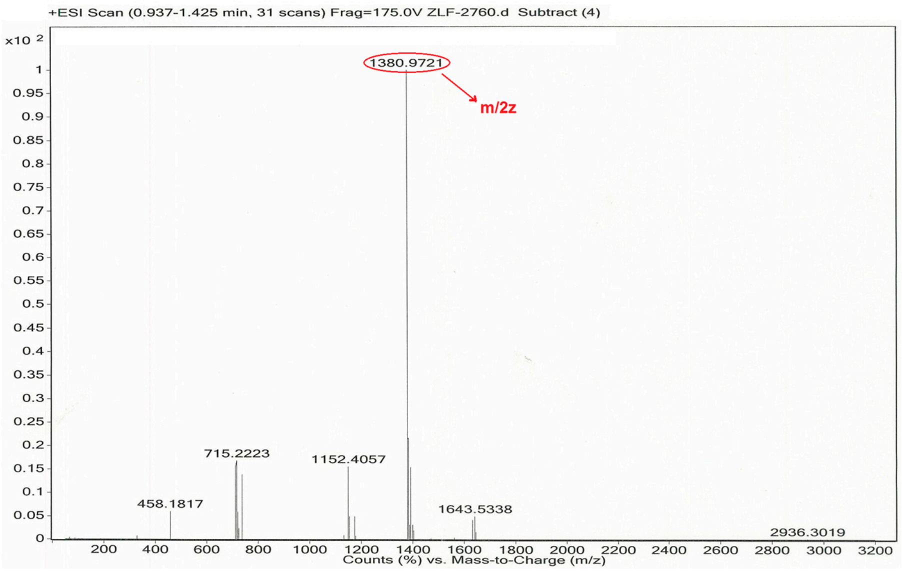

Figure S5. High resolution mass spectra of TPE-triazole-CD.

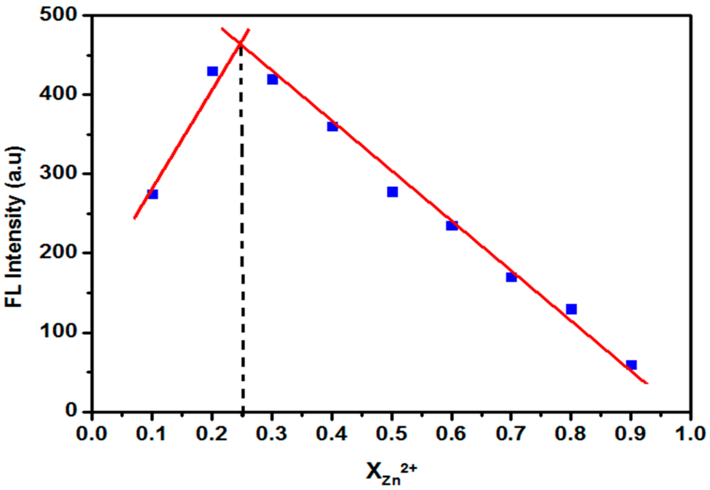

Figure S6. Job's plot for evaluating the chelating ratio of probe to Zn^{2+}. The total concentration of 4 and Zn^{2+} was 50 μM. (λ_{em} = 478 nm).

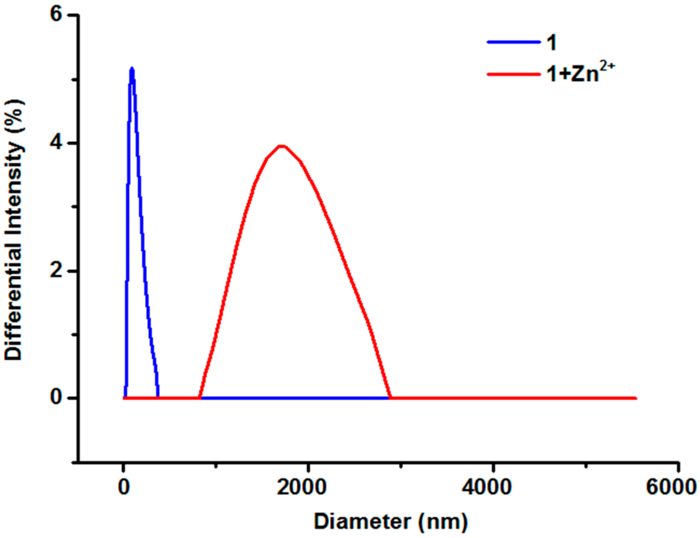

Figure S7. Particle size distribution of TPE-triazole-CD (50 μM) in DMSO/H_2O (1/1, *v*/*v*) solution before and after addition of Zn^{2+} (a quarter equivalent).

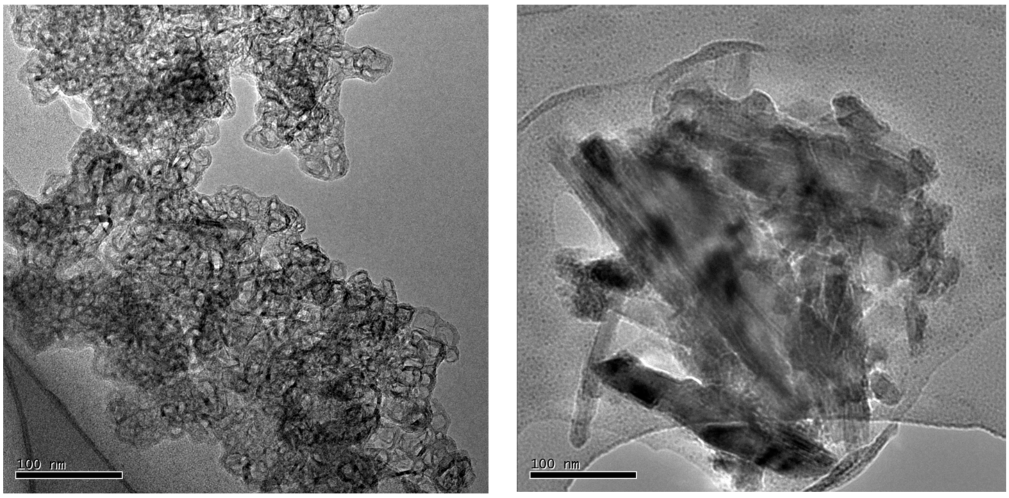

(**a**) (**b**)

Figure S8. TEM images of sensor **1** (100 μM) in DMSO/H_2O (1:1, *v*/*v*) before (**a**) and after (**b**) addition of a quarter equivalent Zn^{2+}.

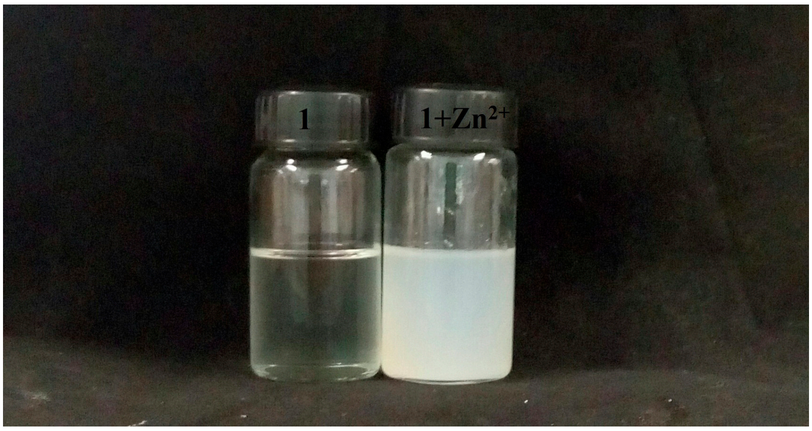

Figure S9. Images of **1** at high concentration in DMSO-H_2O (1/1, *v*/*v*) before and after the addition of Zn^{2+} under daylight.

(a) (b)

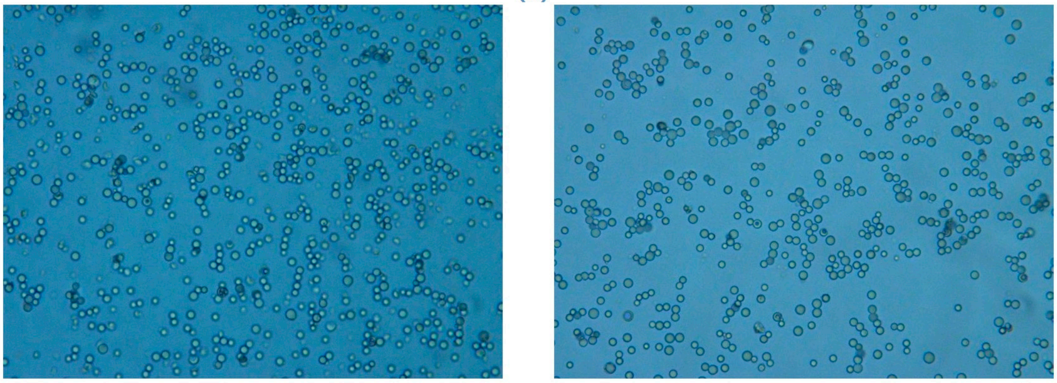

Figure S10. The morphology of silica particles before (**a**) and after (**b**) click reaction.

Table S1. Elemental analysis of alkynl silica and CSP.

Analyte	C	H	N	Surface Loading (μmol·m⁻²)
Alkynl silica	4.88	0.94	0.80	-
CSP	12.31	2.26	1.32	0.58

$$\frac{\mu mol}{m^2} = \frac{(\%C)(10^6)}{(S.A)(n_c)(12.001)}\left[100 - \frac{\%C}{(n_c)(12.001)}(M_r)\right]$$

S.A is the surface area of the silica; n_c is the carbon numbers of N_3-CD; M_r is the molecular weight of N_3-CD.

Table S2. Separation results of **Triazole-CD-CSP**.

Analytes	k_1	α	R_s	*Separation Conditions*
**Dansyl amino leucine**	3.73	2.01	5.44	1% TEAA buffer (pH 4.11)/MeOH (50/50 *v*/*v*)
**Dansyl amino methionine**	2.07	1.49	2.62	1% TEAA buffer (pH 4.11)/MeOH (50/50 *v*/*v*)
**Dansyl aminophenylalanine**	2.84	1.55	2.41	1% TEAA buffer (pH 4.11)/MeOH (50/50 *v*/*v*)
**Dansyl amino serine**	1.66	1.27	1.40	1% TEAA buffer (pH 4.11)/MeOH (50/50 *v*/*v*)
**Flavanone**	1.10	1.61	2.66	ACN/H_2O (40/60 *v*/*v*)
**4′-hydroxyflavanone**	1.12	1.98	3.19	ACN/H_2O (40/60 *v*/*v*)
**6-methoxyflavanone**	0.79	1.00	0	ACN/H_2O (40/60 *v*/*v*)

* flow rate = 0.8 mL·min⁻¹.
